# Data on metal accumulation in the tails of the lizard *Microlophus atacamensis* in a coastal zone of the Atacama Desert, northern Chile: A non-destructive biomonitoring tool for heavy metal pollution

**DOI:** 10.1016/j.dib.2020.106032

**Published:** 2020-07-18

**Authors:** Yery Marambio-Alfaro, Jorge Valdés Saavedra, Luis Ñacari Enciso, Américo López Marras, Antonio E. Serrano, Rodrigo Martínez Peláez, Alexis Castillo Bruna, Gabriel Álvarez Ávalos, Marcela Vidal Maldonado

**Affiliations:** a Applied Sciences – Coastal Marine Systems, Faculty of Marine Sciences and Biological Resources, University of Antofagasta, 2800 Universidad de Antofagasta Av., Antofagasta, Chile; b Parménides Ltda., Zuiderster 1025, Caldera, Atacama, Chile; c Alexander von Humboldt Institute of Natural Sciences, University of Antofagasta, 2800 Universidad de Antofagasta Av., Chile; d Independent Researcher, Chile; e Department of Mathematics, Faculty of Basic Sciences, University of Antofagasta, Antofagasta 1240000, Chile; f Research Center for Advanced Studies of Maule, Vice-Rector's Office for Research and Postgraduate Studies, Catholic University of Maule, San Miguel Campus, San Miguel Av., Talca, Chile; g Department of Engineering in Geo measuring and Geomatics, 2800 Universidad de Antofagasta Av., Chile; h Department of Basic Sciences, Faculty of Sciences, University of Bío-Bío, Chillán, Chile

**Keywords:** Environmental pollution, Heavy metals, Atacama Desert, Bioaccumulation, Lizard

## Abstract

In this data article, we investigated the accumulation of heavy metals in the lizard *Microlophus atacamensis*, in three coastal areas of the Atacama Desert, northern Chile. We captured lizards in a non-intervened area (Parque Nacional Pan de Azucar, PAZ), an area of mining impact (Caleta Palitos, PAL) and an active industrial zone (Puerto de Caldera, CAL). Our methods included a non-lethal sampling of lizard's tails obtained by autotomy. The concentrations of lead, copper, nickel, zinc and cadmium were measured in both soil and prey and compared to those recorded in the lizards' tails. We estimated metal concentrations in the soil, in putative prey and *M. atacamensis* tails, using atomic absorption spectrophotometry. In order to characterize the trophic ecology of *M. atacamensis* and to relate it to possible differences in metal loads between sites, we included a few slaughtered animals to perform a stomach contents analysis (SCA). The software R Core Team (2019) was used to carry out all statistical tests to evaluate and analyze the data, applying a priori and a posteriori statistical tests to test the variance and mean hypotheses. Analysis of the data of the content of heavy metals in the tails, prey and soil inhabited by *M. atacamensis* in PAZ, PAL and CAL showed that the concentration of metals found in the tails and the range of environmental exposure to heavy metals of these animals were related. This article shows for the first time a quantification of the metal concentration on lizard tissues with a non-lethal technique in anthropically disturbed sites in the South Pacific.

## Graphical abstract

**Figure:**
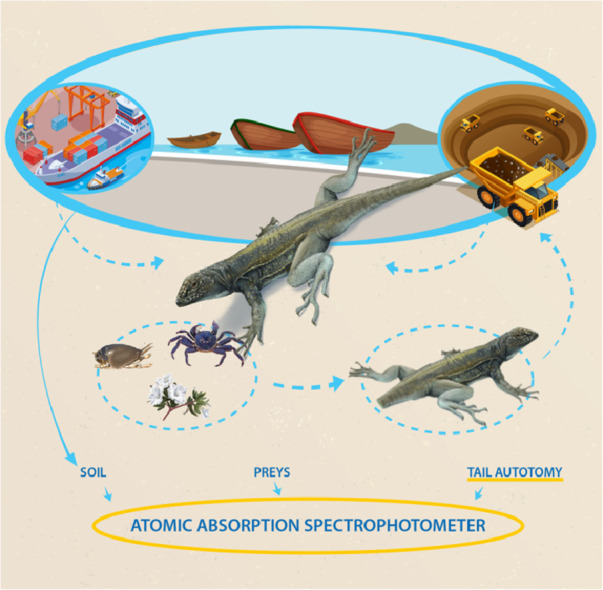

**Specifications Table**

**Table:** 

**Subject**	Environmental Science
**Specific subject area**	Environmental Chemistry
**Type of data**	Image, image, Tables, in excel file (.xlxs)
**How data were acquired**	Atomic absorption spectrophotometer (Shimadzu AA-6300) by flame technique
**Data format**	Raw data, Analyzed
**Parameters for data collection**	Field collection of soil, putative preys and tails of *Microlophus atacamensis* along the coastal desert of Atacama on three sites with different degrees of anthropogenic intervention.
**Description of data collection**	A total of 28 soil, 29 putative preys and 73 tail samples were collected from areas with different degrees of anthropogenic intervention. To leave no doubt that there was no contamination from the used instruments in the sampling process, we have used non-metal instruments. The locations were registered using GPS and the map is provided. Soil samples were collected at a depth of 10-20 cm, the putative preys were obtained manually as well as the tails of lizards.
**Data source location**	1) a) Parque Nacional Pan de Azucar (PAZ, 26° 08′ 59" S 70° 39′ 02" W) b) Caleta Palitos (PAL, 26° 16′ 29" S 70° 39′ 36" W)c) Puerto de Caldera (CAL, 27° 04′ 00" S 70° 49′ 00" W). 1) Atacama Region 2) Chile
**Data accessibility**	Included in the article
**Related research article**	Microlophus atacamensis as a biomonitor of coastal contamination in the Atacama Desert, Chile: an evaluation through a non-lethal technique Yery Marambio-Alfaro, Jorge Valdés Saavedra, Luis Ñacari Enciso, Américo López Marras, Antonio E. Serrano, Rodrigo Martínez Peláez, Alexis Castillo Bruna, Gabriel Álvarez Ávalos, Marcela Vidal Maldonado. ENVPOL_2020_2010 _R1 (in revision)[1]

**Value of the Data**

• Knowledge of metals present in the soil, putative preys and lizard tails provides an essential tool for distinguishing between the contribution of these metals from natural sources and the impact of anthropogenic sources from the coastal desert of Atacama (Northern Chile).
• The data presented will allow an interdisciplinary interpretation of the environmental damage caused by anthropogenic processes.
• The data are unique, but reproducible to the same sites studied or it can be used as a framework for other anthropically disturbed areas.
• These data can be used as a supportive tool for decision makers in regulatory bodies related to industrial fields and it can be used to examine any dynamics or changes in the future.
• The data shows quantification of the degrees of contamination using a non-destructive or non-lethal technique.

## Data description

The Atacama Desert, in Northern Chile, is one of the oldest deserts of the planet and has been arid to semi-arid for millions of years. It is one of the richest territories in the world in terms of porphyry copper deposits, whose heavy mining industry generates waste that significantly affects environmental sustainability.

In this article, we present collected data from January 2017 to November 2018 from three sites, a coastal cove with a well-known legacy of mine tailing discharge (Caleta Palitos, PAL), an active industrial city port (Caldera, CAL) and a National Park (Pan de Azucar, PAZ), spanning about 130 km of a coastal transect of the Atacama Desert (Table 1).

**Table 1:** Heavy metal concentrations (mg kg^−1^) in tails, putative preys and soils from PAZ, PAL and CAL areas of the Atacama Desert, northern Chile

Lizard tails	Site	Taxa	Length	Weight	S-V-L	Sex	Pb	Cu	Ni	Zn	Cd
*Tail*	*PAZ*	Lizard	22.20	31.92	9.50	female	5.24	32.58	14.10	19.28	0.60
*Tail*	*PAZ*	Lizard	26.00	75.00	12.40	male	5.81	29.28	6.34	21.88	0.40
*Tail*	*PAZ*	Lizard	22.50	59.42	12.00	male	5.47	51.79	11.99	9.03	0.85
*Tail*	*PAZ*	Lizard	30.50	76.00	12.50	male	8.14	66.22	31.50	7.20	1.50
*Tail*	*PAZ*	Lizard	30.80	60.82	12.50	male	2.09	38.40	16.25	26.42	0.53
*Tail*	*PAZ*	Lizard	26.00	69.10	12.60	male	3.95	51.89	21.11	26.82	0.74
*Tail*	*PAZ*	Lizard	21.60	26.03	9.30	female	4.48	63.76	22.07	30.59	0.98
*Tail*	*PAZ*	Lizard	24.70	37.95	9.50	female	23.69	20.69	16.10	13.32	1.14
*Tail*	*PAZ*	Lizard	20.80	24.82	8.70	female	25.52	27.50	17.84	15.41	1.07
*Tail*	*PAZ*	Lizard	26.50	77.81	13.00	male	13.59	16.12	17.66	15.55	0.58
*Tail*	*PAZ*	Lizard	24.80	35.00	10.00	female	43.56	31.59	9.09	11.15	0.30
*Tail*	*PAZ*	Lizard	28.80	74.90	10.00	male	32.57	27.06	7.85	9.65	0.22
*Tail*	*PAZ*	Lizard	20.00	23.14	9.00	female	40.31	53.30	11.07	17.77	0.61
*Tail*	*PAZ*	Lizard	15.00	9.55	7.00	female	87.08	103.14	14.72	56.06	1.59
*Tail*	*PAZ*	Lizard	14.40	10.75	7.00	female	88.89	110.24	7.16	42.52	1.84
*Tail*	*PAZ*	Lizard	20.50	14.60	8.00	female	76.14	75.22	6.97	46.03	2.65
*Tail*	*PAZ*	Lizard	14.00	8.00	6.00	female	130.63	130.49	80.89	79.38	3.07
*Tail*	*PAZ*	Lizard	20.10	19.80	8.50	male	46.95	31.77	12.93	22.25	0.47
*Tail*	*PAZ*	Lizard	16.00	20.50	9.00	female	51.67	31.17	17.41	5.77	1.37
*Tail*	*PAZ*	Lizard	25.00	34.36	10.00	female	45.43	39.34	7.22	3.24	1.48
*Tail*	*PAZ*	Lizard	20.30	31.38	10.00	female	63.70	54.97	4.35	31.05	1.82
*Tail*	*PAZ*	Lizard	33.00	60.30	13.00	male	74.01	33.02	2.98	19.52	1.58
*Tail*	*PAZ*	Lizard	14.00	10.15	6.20	female	179.28	49.19	18.36	48.09	5.27
*Tail*	*PAZ*	Lizard	28.00	75.60	13.60	male	82.73	45.78	4.35	13.53	1.80
*Tail*	*PAZ*	Lizard	26.50	84.50	13.20	male	180.38	70.20	9.78	29.04	5.77
*Tail*	*PAZ*	Lizard	28.00	98.20	13.30	male	91.78	54.21	9.00	20.65	2.15
*Tail*	*PAZ*	Lizard	24.50	57.03	12.00	male	153.84	32.53	8.50	49.16	5.28
*Tail*	*PAZ*	Lizard	18.20	17.25	8.00	female	115.79	70.46	5.25	34.18	2.84
*Tail*	*PAZ*	Lizard	27.00	115.60	13.20	male	28.58	70.46	3.00	9.52	1.13
*Tail*	*PAZ*	Lizard	28.20	103.00	13.00	male	28.58	70.46	3.00	9.52	1.13
*Tail*	*CAL*	Lizard	21.00	29.97	10.00	male	22.70	66.67	4.62	21.25	2.22
*Tail*	*CAL*	Lizard	16.00	10.69	9.00	female	33.82	54.38	12.13	30.41	2.61
*Tail*	*CAL*	Lizard	19.00	17.37	11.00	female	36.27	45.20	11.91	22.78	1.49
*Tail*	*CAL*	Lizard	21.00	40.26	10.50	male	35.02	45.94	13.33	36.84	1.68
*Tail*	*CAL*	Lizard	16.60	24.89	9.00	female	33.33	42.10	12.37	38.63	1.69
*Tail*	*CAL*	Lizard	18.00	12.52	6.50	female	17.88	47.53	12.41	34.06	1.86
*Tail*	*CAL*	Lizard	29.00	87.30	13.40	male	3.44	28.60	4.68	14.71	0.91
*Tail*	*CAL*	Lizard	19.80	43.01	11.00	male	94.63	25.63	11.28	39.02	11.21
*Tail*	*CAL*	Lizard	20.20	29.61	10.00	male	84.00	76.96	8.24	29.32	0.99
*Tail*	*CAL*	Lizard	19.00	13.22	7.50	female	96.08	34.04	5.85	33.80	0.94
*Tail*	*CAL*	Lizard	23.50	23.62	9.00	female	100.34	47.15	5.70	37.10	2.47
*Tail*	*CAL*	Lizard	25.00	38.67	10.00	male	55.85	33.53	7.61	13.82	1.56
*Tail*	*CAL*	Lizard	25.00	26.71	10.00	male	55.63	31.75	5.02	17.21	1.62
*Tail*	*CAL*	Lizard	30.00	69.35	12.50	male	62.25	25.52	4.98	19.58	2.24
*Tail*	*CAL*	Lizard	27.50	71.98	12.80	male	71.38	38.48	7.83	18.84	2.11
*Tail*	*CAL*	Lizard	31.00	72.29	13.00	male	38.52	11.19	1.98	10.33	0.85
*Tail*	*CAL*	Lizard	24.50	29.37	10.00	female	63.93	23.61	2.63	15.58	2.23
*Tail*	*CAL*	Lizard	23.00	32.25	9.50	female	87.72	15.04	6.02	13.08	1.53
*Tail*	*CAL*	Lizard	22.50	27.02	9.00	male	81.11	22.48	3.96	15.34	2.76
*Tail*	*CAL*	Lizard	22.00	30.72	9.30	male	72.29	15.82	3.24	16.72	1.97
*Tail*	*PAL*	Lizard	18.00	10.06	7.30	male	65.31	10.07	5.32	19.29	5.76
*Tail*	*PAL*	Lizard	13.00	5.50	6.00	female	121.11	70.23	23.50	61.77	2.26
*Tail*	*PAL*	Lizard	14.30	6.60	6.20	female	113.58	117.52	21.26	60.41	2.51
*Tail*	*PAL*	Lizard	26.50	40.67	11.00	male	69.68	107.92	12.72	41.78	2.06
*Tail*	*PAL*	Lizard	20.50	36.05	11.00	male	76.49	83.13	19.07	31.92	1.43
*Tail*	*PAL*	Lizard	21.00	20.58	9.50	male	117.39	126.92	25.16	58.64	2.44
*Tail*	*PAL*	Lizard	20.00	31.76	10.00	male	73.69	71.19	14.35	35.73	1.46
*Tail*	*PAL*	Lizard	19.00	24.04	9.50	female	110.36	68.74	21.27	67.16	4.11
*Tail*	*PAL*	Lizard	20.00	12.44	7.00	female	114.46	91.33	21.43	70.37	4.11
*Tail*	*PAL*	Lizard	21.00	19.27	8.00	female	81.33	96.99	14.94	50.43	2.76
*Tail*	*PAL*	Lizard	10.00	13.00	5.00	female	94.08	111.17	24.30	43.00	1.76
*Tail*	*PAL*	Lizard	17.00	18.50	7.00	female	97.46	117.70	27.58	47.21	1.88
*Tail*	*PAL*	Lizard	18.20	16.00	6.50	female	112.95	133.22	30.70	53.97	2.36
*Tail*	*PAL*	Lizard	17.60	17.00	6.00	female	66.61	78.17	17.17	25.74	0.78
*Tail*	*PAL*	Lizard	20.10	25.00	8.00	male	74.87	74.87	19.35	29.81	1.12
*Tail*	*PAL*	Lizard	23.30	33.10	11.00	male	72.46	78.14	17.16	29.38	1.19
*Tail*	*PAL*	Lizard	25.40	29.00	10.00	male	141.17	113.39	31.00	40.12	1.31
*Tail*	*PAL*	Lizard	16.00	21.00	7.50	female	108.58	84.63	23.52	30.76	1.03
*Tail*	*PAL*	Lizard	24.00	35.20	10.00	male	113.97	97.75	24.72	32.36	1.13
*Tail*	*PAL*	Lizard	14.90	18.40	6.50	female	97.57	68.91	19.06	53.95	3.18
*Tail*	*PAL*	Lizard	21.20	23.00	8.00	male	100.75	96.02	18.23	56.33	3.42
*Tail*	*PAL*	Lizard	25.70	26.50	9.00	male	71.80	88.85	16.17	47.63	2.99
Prey	Site	Taxa	Pb	Cu	Ni	Zn	Cd				
*Emerita analoga*	*PAZ*	Crustacea	11.40	24.64	10.04	17.55	1.71				
*Ulva sp.*	*PAZ*	Algae	2.62	9.70	4.18	7.23	0.35				
*Ulva sp.*	*PAZ*	Algae	1.21	7.01	2.98	6.00	0.21				
*Brown algae*	*PAZ*	Algae	84.82	53.36	14.59	32.27	2.38				
*Flowers 1*	*PAZ*	Flora	100.23	49.72	14.21	25.38	1.85				
*Flowers 2*	*PAZ*	Flora	78.61	30.18	12.76	31.85	0.75				
*Amphipods*	*PAZ*	Crustacea	10.57	6.26	15.76	62.87	6.18				
Small crab 1	*PAZ*	Crustacea	1.23	34.74	3.04	3.00	0.50				
*Small crab 2*	*PAZ*	Crustacea	22.82	75.60	8.06	38.42	66.00				
*Echinolittorina peruviana*	*CAL*	Molusca	107.91	28.33	5.67	28.55	2.19				
*Echinolittorina peruviana*	*CAL*	Molusca	94.80	40.85	4.29	23.97	1.55				
*Echinolittorina peruviana*	*CAL*	Molusca	94.09	35.80	2.29	34.75	1.49				
*Echinolittorina peruviana*	*CAL*	Molusca	138.36	77.02	0.71	53.65	1.66				
*Flowers*	*CAL*	Flowers	8.92	3.27	0.03	3.25	0.18				
*Small crab 1*	*CAL*	Crustacea	64.57	19.91	2.55	16.06	0.70				
*Small crab 2*	*CAL*	Crustacea	161.27	38.66	8.04	41.14	1.78				
*Small crab 3*	*CAL*	Crustacea	19.63	25.74	4.23	21.64	1.21				
*Brown algae*	*CAL*	Algae	5.41	17.56	0.90	1.74	0.38				
*Colpomenia sp.*	*CAL*	Algae	5.58	36.74	0.85	2.01	0.49				
*Glossophora Kuntii*	*CAL*	Algae	2.96	4.82	0.15	0.75	0.19				
*Tenebronidae*	*CAL*	Insecta	46.83	8.11	3.55	12.32	2.66				
*Tenebronidae*	*CAL*	Insecta	64.54	43.84	14.12	18.21	1.95				
*Brown algae*	*CAL*	Algae	64.64	32.76	10.44	52.04	1.19				
*Echinolittorina peruviana*	*PAL*	Molusca	41.85	49.16	2.82	10.33	2.18				
*Echinolittorina peruviana*	*PAL*	Molusca	27.21	34.41	1.79	13.98	2.90				
*Echinolittorina peruviana*	*PAL*	Molusca	26.35	41.85	1.42	7.17	1.58				
*Flowers 1*	*PAL*	Flowers	7.51	6.76	4.51	1.64	0.69				
*Flowers 2*	*PAL*	Flowers	88.51	48.70	13.02	1.33	31.76				
*Algae*	*PAL*	Algae	49.67	23.35	14.57	10.44	2.47				
*Residue*	*PAL*	Residue mix	59.59	40.17	10.51	45.09	0.83				
Soil	Site	Pb	Cu	Ni	Zn	Cd					
Soil	*PAZ*	23.27	30.34	15.60	8.30	8.23					
Soil	*PAZ*	21.58	31.25	15.68	8.90	8.82					
Soil	*PAZ*	10.71	26.04	12.34	13.32	8.10					
Soil	*PAZ*	8.22	23.28	12.22	11.24	7.72					
Soil	*PAZ*	24.36	32.83	16.46	9.18	9.19					
Soil	*PAZ*	8.80	24.62	13.28	9.93	8.66					
Soil	*PAZ*	13.89	32.50	18.00	7.61	9.67					
Soil	*PAZ*	14.08	43.56	20.14	19.11	14.67					
Soil	*CAL*	25.57	31.20	16.33	8.44	7.89					
Soil	*CAL*	14.37	31.15	15.13	7.00	9.89					
Soil	*CAL*	13.76	30.32	15.35	7.65	9.05					
Soil	*CAL*	16.85	32.10	15.97	9.28	9.06					
Soil	*CAL*	9.69	32.59	14.02	10.52	8.65					
Soil	*CAL*	9.52	26.69	13.92	13.34	11.00					
Soil	*CAL*	10.94	26.37	12.82	13.73	9.05					
Soil	*CAL*	7.62	27.31	14.03	10.79	9.01					
Soil	*PAL*	25.38	35.65	15.84	7.14	7.07					
Soil	*PAL*	28.30	38.81	17.45	7.65	7.58					
Soil	*PAL*	29.53	38.92	17.22	8.01	7.83					
Soil	*PAL*	24.13	32.86	16.34	8.99	9.07					
Soil	*PAL*	16.09	31.37	15.37	9.67	9.77					
Soil	*PAL*	15.06	31.69	14.98	7.86	10.16					
Soil	*PAL*	13.48	30.15	14.31	7.23	9.41					
Soil	*PAL*	14.13	32.08	16.06	7.60	9.50					
Soil	*PAL*	11.84	29.51	14.53	8.36	9.01					
Soil	*PAL*	9.56	26.04	13.19	9.64	7.90					
Soil	*PAL*	8.89	30.49	14.86	12.52	9.80					
Soil	*PAL*	9.02	25.65	12.67	12.28	10.81					

**Fig. 1:**
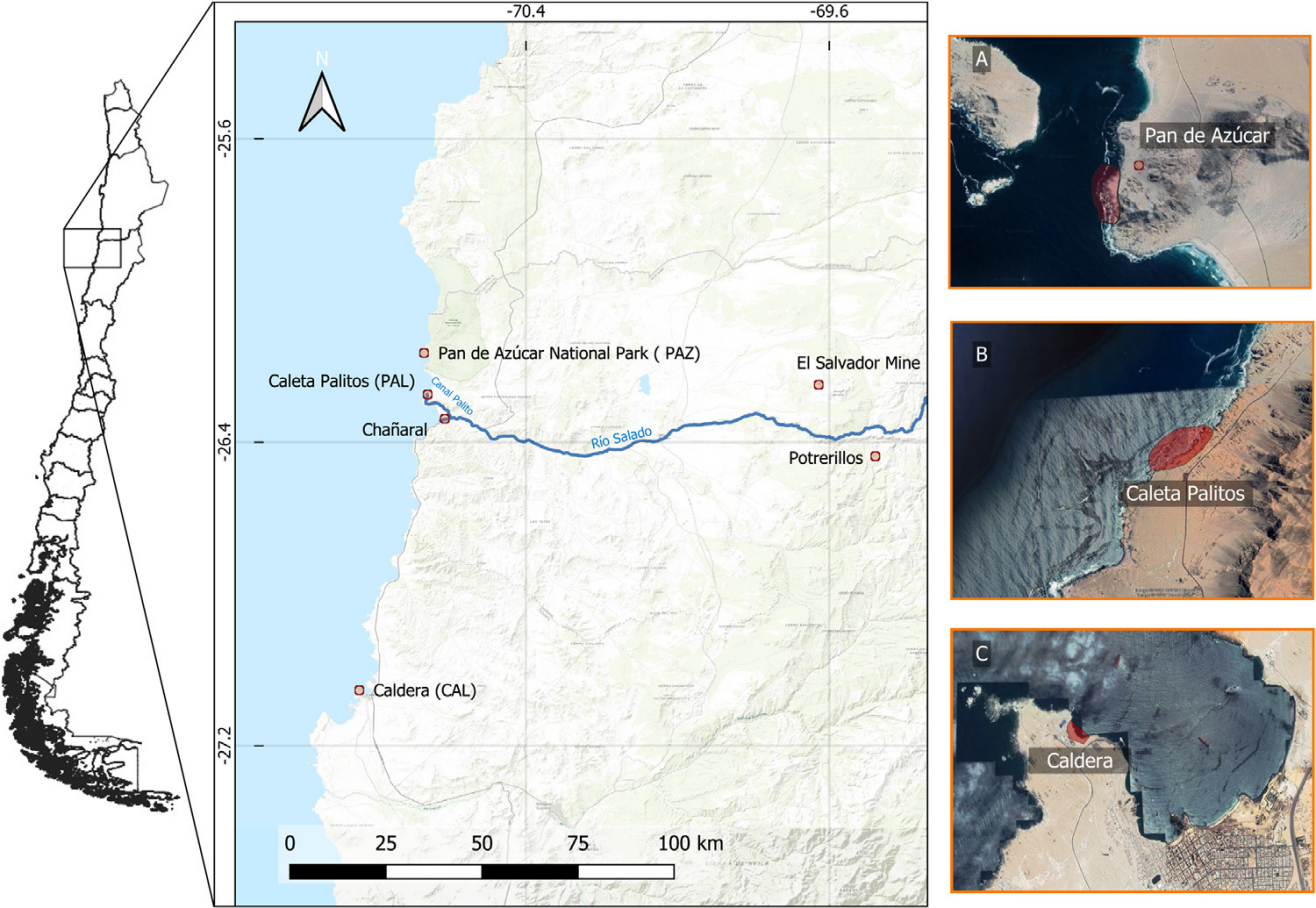
Aerial view of the sites sampled is shown relative to a map of South America. The three sampling sites from North to South are Parque Nacional Pan de Azucar (A, PAZ), Caleta Palitos (B, PAL) and Puerto de Caldera (C, CAL). The target taxon *M. atacamensis* is primarily present in the intertidal zone. These images correspond to a mosaic generated using Google Maps-Digital Globe Company. The images are native 30 cm resolution imagery. The average position of these images is 5m CE90 in lat/long.

*Soil:* We obtained a total of 28 samples to determine the metal content in soils of the studied sites. The samples were stored in plastic bags previously treated with HCl (1M). Considering the same sampling transect line lizards were also captured (Fig. 1).

*Prey:* 29 putative preys were obtained manually at the three sites using hand searches and, where necessary (e.g. for flying insects), using hand nets. Samples were returned to the laboratory, identified, and where necessary soft tissues were removed from inorganic carapaces (decapods) or shells (mollusks). Samples were then dried (60°C for 48 h) before processing for subsequent analysis for metal concentrations.

*Tails:* A total of 72 adult *M. atacamensis* lizards (CAL *n*=20, PAL *n*= 22, PAZ *n*= 30) (Table 1) were captured randomly within five meters of each side of an imaginary transect during the hottest hours of the day (11:00–15:00 h) [2]. We captured each animal carefully using a rod with a sliding lasso in order to preserve their original tails, ensuring that the process of autotomy had not taken place [3].

Subsequently, in the laboratory the collected individuals were sexed, measured and weighed [4,5]. All individuals demonstrated autotomy of their tails; thus, there was no need to remove them surgically. After sacrificing 27 lizards, their soft parts (stomach, lungs, liver, heart and kidney) were dissected out. Finally, after measuring tissues weight, we stored the tails and soft tissues in sterile vials for subsequent processing and analysis for heavy metals.

## Experimental design, materials, and methods

*Stomach content:* Twenty-seven *M. atacamensis* from the three sites studied (CAL *n*=10, PAZ *n* =10, PAL *n* =7) were dissected. The stomach content samples were returned to the laboratory, identified, and when necessary, soft tissues were removed from inorganic carapaces (decapods), shells (mollusks) or flowers. The stomach contents were observed under a dissection microscope and identified to the highest possible taxonomic resolution supported by a series of keys and identification guides [6], [7], [8], [9]. The total blotted wet mass of each prey category was estimated to ± 0.001 g. We determined the relative importance of each prey to the diet of *M. atacamensis* by calculating the frequency of occurrence (FO) and the percentage contribution by mass (%M) [10] (Table 2).

**Table 2:** Preys for each evaluated site, frequency determined in the stomachs of slaughtered animals. n = number of animals slaughtered per site in PAZ, PAL and CAL.

	Site																											
Prey item (%)	Caldera (CAL)										Pan de Azucar (PAZ)											Palito (PAL)						
Amphipod	0	0	0	0	0	40	0	10	0	0	0	10	0	0	55	0	15	10	0	0	0		0	0	0	0	0	0
Decapod	0	13	0	0	0	0	0	0	0	0	0	0	0	15	0	0	0	0	0	35	0		0	0	10	0	0	0
Echinolittorina sp.	0	0	0	0	0	0	0	0	0	10	0	0	0	0	0	0	0	0	0	0	0		0	0	10	0	0	10
Ulva sp.	0	0	0	0	0	50	0	70	100	30	60	0	70	65	0	0	80	20	100	15	60		30	20	0	60	10	0
Porphyra sp.	0	0	0	0	0	0	0	0	0	0	0	65	0	0	0	0	0	0	0	45	0		0	0	0	0	0	0
UID insecta	0	0	10	0	0	10	100	0	0	0	0	0	0	0	0	0	0	0	0	0	0		0	0	0	0	0	20
UID Lepidoptera	0	25	0	0	5	0	0	0	0	0	0	0	0	0	0	0	0	0	0	0	0		0	0	0	0	0	0
UID diptera	10	62	90	0	10	0	0	0	0	0	0	0	0	15	0	0	0	0	0	0	10		10	10	20	10	90	40
UID Coleptera	0	0	0	100	0	0	0	0	0	0	0	0	0	0	0	0	0	0	0	0	0		0	0	0	0	0	0
Tenebrionidae	0	0	0	0	0	0	0	0	0	0	10	0	20	0	0	0	0	0	0	0	0		0	0	0	0	0	0
*Microlophus atacamensis*	0	0	0	0	5	0	0	0	0	0	0	0	0	0	0	90	0	0	0	0	0		0	0	0	0	0	0
Flowers	90	0	0	0	80	0	0	20	0	60	0	0	0	0	0	0	0	0	0	0	30		60	70	60	30	0	30
Fish	0	0	0	0	0	0	0	0	0	0	0	20	0	0	0	0	0	0	0	0	0		0	0	0	0	0	0
Sand	0	0	0	0	0	0	0	0	0	0	30	5	10	5	45	10	5	70	0	5	0		0	0	0	0	0	0
Total (%)	100	100	100	100	100	100	100	100	100	100	100	100	100	100	100	100	100	100	100	100	100		100	100	100	100	100	100

*Heavy metals (Lead, Copper, Nickel, Zinc and Cadmium):* For the quantification of metals per site the methodology described by Castillo and Valdés [11] was followed for the analytical pre-treatment on putative preys and tails (Table 1). The content of metals in soil was measured in the fraction <63 μm, after drying the samples at 40°C. For this, between 0.2 and 0.6 g of dry soil was disaggregated in a MARS-X microwave digester (CEM model 350) with a mixture 12 ml of HNO3:HCl (3: 1 ratio) at 150°C for 20 min according to the US- EPA 3051A procedure (EPA, 2007). Finally, the resulting solution was filtered with a 0.45 μm filter and diluted to 25 ml with deionized water [12]**.**

The soft tissues were separated and homogenized in an agate mortar for biological material until a wet paste was obtained. Subsequently, between 0.5 and 1.0 g of sample was added in a Teflon beaker with 10 ml of HNO_3_ (Suprapur, Merck®) and was disintegrated into a microwave digester (MARS-5), according to the US-EPA procedure 3051A (digestion at 180°C for 10 minutes). Finally, the resulting solution was diluted to 25 ml with deionized water.

The analysis of Pb, Cu, Ni, Zn and Cd from organisms and soil was performed with an atomic absorption spectrophotometer (Shimadzu AA-6300) by flame technique. The analytical procedure was checked using the certified standard reference material DORM-3 and MESS-3 (National Research Council, Canada). The analytical error was less than 5% and the results were expressed as mg kg^–1^ (Table 3).

**Table 3:** Indices used in this report and their respective formulas, parameters, descriptions and interpretations classes.

Indices	Used formula	Parameters	Description	Interpretation
BAF Bioaccumulation Factor	(*C_biota_*, mg kg^−1^)/ (*C_soil_,* mg kg^−1^)	Concentration detected in the lizard tails (*C_biota_*, mg kg^−1^), concentration of the metal measured from the soil (*C_soil_,* mg kg^−1^)	It was calculated dividing the metal concentration detected in the lizard tails by the concentration of the metal measured from the soil	values >1. A value greater than 1 implies bioaccumulation with respect to the reference environmental matrix
TTF	**(C** organism's tissue mg kg^−1^)/ (***C****organism’ food* mg kg^−1^)	**C** organism's tissue, is metal concentration in the organism's tissue, ***C****organism’ food* mg kg^−1^ is metal concentration in the organism's food.	It was calculated dividing metal concentration in the organism's tissue / Metal concentration in the organism's food.	A TTF value >1 indicates a possibility of biomagnification, while values <1 suggest that biomagnification is unlikely. For the TTF calculations, we considered a range of assimilation efficiencies and ingestion rates for all organisms
RI *Potential Ecological Risk*	$\begin{array}{c} RI=\sum_{1=1}^{n}E_{r}^{1}\\ E_{r}^{I}=T_{r}^{1}=\frac{C_{i}^{1}}{C_{r}^{1}} \end{array}$	where *T_r_* is the toxic response factor for a specific heavy metal, this factor was 30, 5, 5, 5, and 1 for Cd, Cu, Ni, Pb, and Zn, respectively. *C_i_* is the metal concentration, *C_r_* is the background value of heavy metal in soil *E_r_* is the individual potential ecological risk factor	*RI* is a composite index that indicates the potential ecological risk of total heavy metals in soils, and *n* is the total number of the estimated heavy metals	*RI*<150 Low Risk 150<*RI*<300 Moderate Risk 300<*RI*<600 Considerable Risk *RI*>600 High Risk

*Calculation of the Bioaccumulation Factor (BAF), Potential Ecological Risk (RI), and Trophic Transfer Factor (TTF):* The BAF was calculated dividing the metal concentration detected in the lizard tails (*C_biota_*, mg kg^−1^) by the concentration of the metal measured in the sediment (*C_soil_*, mg kg^−1^, Table 4).

**Table 4:** . BAF of metals in the three sites studied. Values greater than 1 imply that there is bioaccumulation with respect to the reference environmental matrix. Bioaccumulation factors (*C_biota_*, mg kg^−1^)/(*C_soil_,* mg kg^−1^) higher than 1 are shown in bold.

Sites	Pb	Cu	Ni	Zn	Cd
PAZ	**3.71**	**1.72**	0.90	**2.26**	0.18
PAL	**5.56**	**2.82**	**1.33**	**5.03**	0.25
CAL	**4.23**	**1.23**	0.49	**2.36**	0.24

The RI of total heavy metals toxicity was calculated using Eq. (1) [13].

(1) $$\begin{array}{c} RI=\sum_{1=1}^{n}E_{r}^{1}\\ E_{r}^{I}=T_{r}^{1}=\frac{C_{i}^{1}}{C_{r}^{1}} \end{array}$$

In Eq. (1), where *T_r_* is the toxic response factor for a specific heavy metal, this factor was 30, 5, 5, 5, and 1 for Cd, Cu, Ni, Pb, and Zn respectively. *C_i_* is the metal concentration in the samples, *C_r_* is the background value of heavy metal in soil (Table 5) [14], *E_r_* is the individual potential ecological risk factor, *RI* is a composite index that indicates the potential ecological risk of total heavy metals in soils, and *n* is the total number of the estimated heavy metals (Table 6).

**Table 5:** . Background soil concentrations expressed in mg kg^–1^.

Authors	Pb	Cu	Ni	Zn	Cd
Background values^a^	12.7 mg kg^–1^	91.6 mg kg^–1^	41.7 mg kg^–1^	75.9 mg kg^–1^	1.2 mg kg^–1^
Background values^b^	32 mg kg^–1^	18,5 mg kg^–1^	20 mg kg^–1^	64 mg kg^–1^	1 mg kg^–1^

^a^Cenma 2014, ^b^Background values of world soils (Alloway 1995).

**Table 6:** . Ecological risk index values RI for PAZ, PAL and CAL, show moderate risk for all sites studied.

RI for sites	RI	Type of Risk
PAZ	296.8	Moderate Risk
PAL	285.6	Moderate Risk
CAL	290.6	Moderate Risk

*Calculation of Trophic Transfer Factor (TTF):* It is calculated dividing the metal concentration in the organism's tissue by the metal concentration in the organism's food [15]. A TTF value >1 indicates a possibility of biomagnification, while values <1 suggest that biomagnification is unlikely. For the TTF calculations, we considered a range of assimilation efficiencies and ingestion rates for all organisms (Table 7). Rearranging this equation to express the ratio of metal concentration in an organism to the concentration in its prey allows an assessment of the potential of a particular metal to biomagnify at different sequential steps in the food chain.

**Table 7:** . TTF of metals from prey to lizard tissue in the three sites studied. A TTF value >1 indicates a possibility of biomagnification, while values <1 suggest that biomagnification is unlikely. Values higher than 1 are shown in bold.

Sites	Pb	Cu	Ni	Zn	Cd
PAZ	**1.66**	**1.63**	**1.62**	0.99	0.19
PAL	**2.22**	**2.59**	**2.93**	**3.49**	0.38
CAL	0.91	**1.24**	**1.76**	**1.08**	**1.79**

## Declaration of Competing Interest

The authors declare that they have no known competing financial interests or personal relationships which have, or could be perceived to have, influenced the work reported in this article.

## Appendix Supplementary materials

Image, application 1

Image, application 2
